# Immune deficiency due to SARS-CoV-2 infection in a child with GATA2-mutated AML: A case report

**DOI:** 10.1097/MD.0000000000043734

**Published:** 2025-08-08

**Authors:** Jiale Cheng, Chengzhu Liu, Songji Tu, Jinhua Chu, Linhai Yang, Lingling Huang, Huaju Cai, Zhengyu Wu, Anbang Wei, Yi Hong, Zhiwei Xie, Ningling Wang, Kunlong Zhang

**Affiliations:** a Department of Paediatrics, The Second Affiliated Hospital of Anhui Medical University, Hefei City, China.

**Keywords:** acute myeloid leukaemia, children, GATA2, immunodeficiency, SARS-CoV-2

## Abstract

**Rationale:**

Mutations in the guanine-adenine-thymine-adenine 2 (GATA2) gene can lead to immunodeficiency and haematological diseases, including acute myeloid leukaemia (AML). Severe acute respiratory syndrome coronavirus 2 (SARS-CoV-2) infection has been reported to impair immune function, but its effects on GATA2 mutation carriers remain unclear. This study reports a rare case of persistent immunodeficiency in a child with AML and GATA2 mutation after SARS-CoV-2 infection, emphasizing the role of viral infection in immune dysfunction in such patients.

**Patient concerns:**

A 9-year-old AML patient developed fever, cough, persistent immunodeficiency, and recurrent severe infections after SARS-CoV-2 infection in haematological remission.

**Diagnoses:**

The patient was diagnosed with AML-M2 with germline GATA2 mutation. After chemotherapy, he achieved haematological remission. After SARS-CoV-2 infection, he showed significant immunodeficiency manifestations and recurrent infections.

**Interventions:**

The patient received combined chemotherapy based on CALSIII-AML18 and achieved haematological remission. After SARS-CoV-2 infection, comprehensive treatments – for example, antiviral (Paxlovid), antibacterial, antifungal, and hormonal therapies – were used to support immune function. The patient completed HLA matching for allogeneic hematopoietic stem cell transplantation and is scheduled to undergo transplantation.

**Outcomes:**

Despite various immune support treatments after SARS-CoV-2 infection, the patient still had persistent immune deficiency and recurrent infections (e.g., pneumonia and hepatitis B). The patient is currently stable and waiting for hematopoietic stem cell transplantation.

**Lessons:**

For AML patients with GATA2 mutation who achieve remission, SARS-CoV-2 infection may still trigger severe and persistent immunodeficiency, leading to serious infections and affecting prognosis. Therefore, early identification of GATA2 mutations and early implementation of hematopoietic stem cell transplantation are key to improving prognoses.

## 1. Introduction

Guanine-adenine-thymine-adenine 2 (GATA2) is a zinc finger protein transcription factor and an important regulator of hematopoiesis. This protein plays a critical role in the homeostasis of hematopoietic stem cells and the development of lymphocytes.^[1]^ Patients with GATA2 mutations present with various clinical manifestations,^[2]^ primarily multiple types of cytopenia, including B-cell, natural killer (NK)-cell, monocyte, and lymphocyte cytopenias; familial myelodysplastic syndrome/acute myeloid leukaemia (AML); susceptibility to immunodeficiency and various infections; pulmonary alveolar proteinosis; and pulmonary hypertension. Identifying GATA2 mutations is important for family screening, rapid diagnosis, bone marrow transplantation treatment, and the selection of potential donors.

We report a case of a child with AML. Upon admission, a comprehensive transcriptome analysis of haematological tumors revealed a GATA2 gene mutation, which was inherited from the patient’s mother and identified as a germline mutation. The patient’s mother had normal immune function and had not developed leukaemia. The patient received chemotherapy under the CALS III-AML18 protocol (Ethics No. PJ-YX2018-041 F1). After 6 months of combined chemotherapy, the patient’s AML remained in continuous haematological remission. Six months after treatment discontinuation, the patient contracted severe acute respiratory syndrome coronavirus 2 (SARS-CoV-2), which caused coronavirus disease 2019, leading to persistent immunodeficiency. This immunodeficiency resulted in repeated and difficult-to-control infections. The patient is currently scheduled to undergo allogeneic hematopoietic stem cell transplantation (HSCT).

## 2. Case presentation

**Figure 1.**
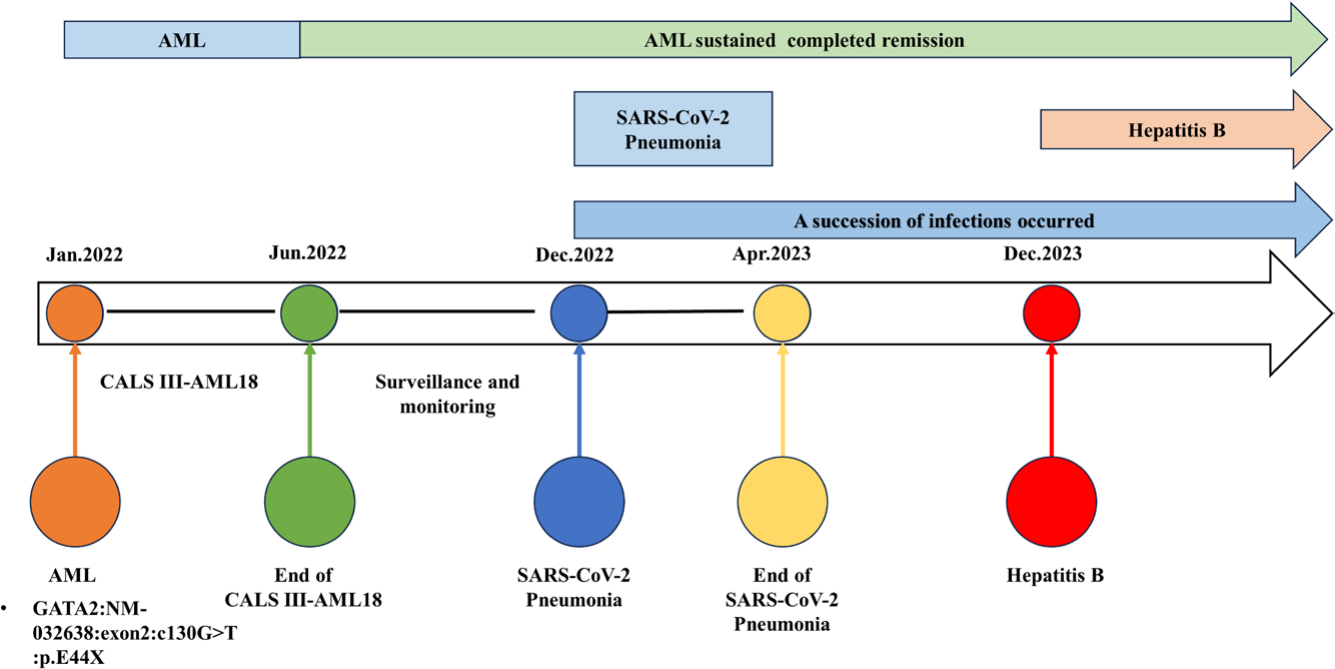
Timeline of events in the case. AML = acute myeloid leukaemia, GATA2 = guanine-adenine-thymine-adenine 2, SARS-CoV-2 = severe acute respiratory syndrome coronavirus 2.

A 9-year-old boy was admitted to our hospital on January 7, 2022, because of pallor and fatigue for 1 month (the timeline of the case report is shown in Fig. 1). Physical examinations at admission revealed pallor and enlarged palpable lymph nodes on both sides of the neck. The liver was located 1.5 cm below the ribs and had a moderate texture, and the spleen was located 1.5 cm below the ribs and had a moderate texture. The complete blood count results were as follows: white blood cell count (WBC), 49.66 × 10^9^/L; monocyte count, 19.55 × 10^9^/L; and lymphocyte count, 8.11 × 10^9^/L. The immunoglobulin levels were as follows: immunoglobulin G, 10.01 g/L; immunoglobulin A, 1.92 g/L; and immunoglobulin M, 0.73 g/L. The peripheral blood lymphocyte subpopulations were as follows: total T-lymphocyte count, 5117/μL; CD4+ T-cell count, 2473/μL; CD8+ T-cell count, 2537/μL; B-cell count, 740/μL; and NK-cell count, 258/μL. Bone marrow cytology revealed that 31% of the cells were blasts, peroxidase stain positive, nonspecial esterase stain positive, and chloroacetate esterase specific stain positive, and suggestive of AML, M2. Bone marrow immunophenotyping revealed that 32.9% of the immature myeloid cells were abnormal. The patient was negative for the bone marrow fusion gene. The bone marrow karyotype was 46,XY[10]. Comprehensive transcriptome sequencing of the bone marrow revealed the following results: GATA2 (NM-032638), exon 2: c130G > T: p.E44X heterozygous mutation, with a frequency of 47.8%, which was considered a germline mutation. Family screening revealed that the father had a wild-type genotype and the mother had a heterozygous mutation, indicating that the mutation was inherited from the mother (the sequencing files were deposited into the NCBI SRA database and can be retrieved at https://www.ncbi.nlm.nih.gov/ accession number PRJNA1139997). Chemotherapy following the CALSIII-AML18 regimen started on January 15, 2022, with MAG*2 → HA → EA → LA. The patient’s bone marrow remained in haematological remission during chemotherapy. Allo-HSCT was recommended to the patient’s parents, but they refused the procedure. After treatment was discontinued on June 20, 2022, follow-ups in July and November 2022 revealed a trend towards the recovery of peripheral blood lymphocyte subpopulations and immunoglobulins, with no infections occurring during this period. The patient’s complete blood count, peripheral blood lymphocyte subpopulation and immunoglobulin test results are shown in Table 1.

**Table 1:** Complete blood count, peripheral blood lymphocyte subpopulation, and immunoglobulin results of the patient over time.

	Complete blood count			Peripheral blood lymphocyte subpopulation					Immunoglobulin			
Date	WBC (4.30–11.30 × 10^9^/L)	Mono# (0.13–0.76 × 10^9^/L)	Lym# (1.50–4.60 × 10^9^/L)	T cell/μL (723–2737)	CD4 + T cell/μL (404–1612)	CD8 + T cell/μL (220–1129)	B cell/μL (80–616)	NK cell/μL (80–724)	IgG g/L (7.00–16.60)	IgA g/L (0.70–3.50)	IgM g/L (0.50–2.60)	Event
1/2022	49.66	19.55	8.11	5117	2473	2537	740	258	10.01	1.92	0.73	AML
7/2022	4.30	0.08	0.82	238	169	62	23	21	3.28	0.21	0.1	$
11/2022	5.38	0.13	1.64	824	466	342	68	54	5.43	0.53	0.38	*
4/2023	9.36	0.02	0.22	192	123	67	0	4	3.17	0.4	0.25	#
6/2023	1.74	0.03	0.38	270	176	94	0	10	6.69	0.27	0.08	*
12/2023	7.40	0.04	0.23	316	169	141	0	7	4.02	0.39	0.09	Hepatitis B
6/2024	3.98	0.01	0.33	324	137	185	0	17	5.66	0.22	0.04	*

AML = acute myeloid leukaemia, IgA = immunoglobulin A, IgG = immunoglobulin G, IgM = immunoglobulin M, Lym# = lymphocytes, Mono# = monocytes, NK = natural killer, WBC= white blood cell.

*, Serial surveillance.

**$**, End of CALS III-AML18.

**#**, treatment discontinuation of severe acute respiratory syndrome coronavirus 2 infection.

On December 28, 2022, the patient was admitted to our hospital because of cough for 1 week and fever for 1 day. After admission, the patient received oxygen inhalation and oxygen saturation monitoring, along with intravenous ceftriaxone (100 mg/kg daily) for 5 days plus the oral compound sulfamethoxazole (25 mg/kg daily, divided into 2 doses and administered 3 d/wk). On the 6th day of hospitalization, the child still had recurrent fever and worsening cough, and wet rales were heard in the lungs. Blood tests revealed the following findings: WBC count, 4.04 × 10⁹/L; neutrophil count, 3.86 × 10⁹/L; monocyte count, 0.01 × 10⁹/L; lymphocyte count, 0.11 × 10⁹/L; hemoglobin level, 119 g/L; platelet count, 247 × 10⁹/L; C-reactive protein level, 102 mg/L; and procalcitonin level, 4.6 ng/mL. A SARS-CoV-2 nucleic acid test was positive, and chest computed tomography indicated bilateral pneumonia. Fibreoptic bronchoscopy was performed, and a clinical diagnosis of mixed pulmonary infection was made. Treatment included a 5-day course of Paxlovid (150 mg nirmatrelvir + 50 mg ritonavir, twice daily, on the basis of the Phase 2/3 EPIC-PEDS study) + intravenous methylprednisolone (2 mg/kg daily) for 7 days + intravenous cefoperazone sulbactam (160 mg/kg daily) for 2 weeks. In the 4th week of hospitalization, the patient developed recurrent fever, cough, and tachypnoea, with a C-reactive protein level of 58 mg/L, a procalcitonin level of 1.8 ng/mL, and a positive *G* test. The treatment regimen was adjusted to intravenous cefoperazone sulbactam (160 mg/kg daily) for 1 week + intravenous amphotericin B (0.6 mg/kg daily) for 2 weeks plus a single dose of intravenous human immunoglobulin (15 g). By the 6th week, the child’s body temperature had normalized, but the cough persisted. Repeated chest computed tomography still revealed bilateral pneumonia, and oral voriconazole (200 mg, q12h) was continued. Two months later, the SARS-CoV-2 nucleic acid test was negative. By the 4th month, the patient had a normal body temperature, no cough, and stable breathing, and repeated chest computed tomography showed basic resolution of bilateral pneumonia, then treatment was subsequently discontinued. The complete blood cell count results were as follows: WBC count, 9.36 × 10^9^/L; monocyte count, 0.02 × 10^9^/L; and lymphocyte count, 0.22 × 10^9^/L. The immunoglobulin levels were as follows: immunoglobulin G, 3.17 g/L; immunoglobulin A, 0.40 g/L; and immunoglobulin M, 0.25 g/L. The peripheral blood lymphocyte subpopulations were as follows: CD4+ T-cell count, 123/μL; CD8+ T-cell count, 67/μL; B-cell count, 0.00/μL; and NK-cell count, 4.00/μL. After SARS-CoV-2 infection, the patient exhibited persistent decreases in lymphocyte, monocyte, CD4+ T-cell, CD8+ T-cell, B-cell, and NK-cell counts and experienced recurrent opportunistic infections. In December 2023, the patient contracted hepatitis B with 3 positive test results and was treated with entecavir (0.015 mg/kg daily). The child has suffered from frequent infections and been hospitalized multiple times for antibiotic anti-infective therapy and immunoglobulin infusion to support immune function. Given that the matching process is complete, the child will soon receive hematopoietic stem cell transplantation (HSCT) treatment.

## 3. Discussion

In this case report, we describe a patient with AML associated with a GATA2 gene mutation. After combination chemotherapy, the patient’s AML remained in complete haematological remission, and during the follow-up period, his immune function was essentially restored, with no infections occurring. However, after contracting SARS-CoV-2, the patient developed persistent immune deficiency and experienced recurrent infections. The c.130G > T (p.Glu44*) nonsense mutation in the GATA2 gene was inherited from the patient’s mother. Interestingly, before being infected with SARS-CoV-2, the patient’s immune function was normal, and his mother did not have immune deficiency or AML.

Mutations in the GATA2 gene can manifest as various clinical presentations, but they are primarily considered immune system disorders and bone marrow abnormalities. The main characteristics of GATA2 deficiency include cytopenia, immunodeficiency, and opportunistic infections, with an increased risk of developing myelodysplastic syndrome and AML.^[3,4]^ Owing to the relative rarity of GATA2 deficiency, its variable clinical presentations, and the lack of specific laboratory tests, early detection can be challenging. GATA2 mutations increase the risk of SARS-CoV-2 infection; furthermore, patients with severe coronavirus disease 2019 may experience cytokine storm syndrome, leading to lymphocyte depletion, impaired host immune function, disease progression, and worsening of the condition.^[1,5–7]^

The patient in this case report remained in sustained haematological remission after treatment but developed persistent immune deficiency after SARS-CoV-2 infection, which led to recurrent respiratory infections as well as hepatitis B infection. Numerous studies have reported that SARS-CoV-2 infection can cause immune deficiency and exacerbate clinical symptoms.^[8–10]^ The patient’s mother had a GATA2 gene mutation but had not developed AML, which suggests significant differences in the age of disease onset and clinical manifestations, even among family members.^[11,12]^ Currently, HSCT remains the only treatment for GATA2 deficiency. Clinically, after a GATA2 gene defect is detected in patients, finding a suitable donor for HSCT as early as possible is essential to avoid delays in treatment. However, this study also has some limitations. This report is based on only 1 child and cannot be generalized to all children with AML carrying GATA2 mutations. There may be significant differences in immune responses to SARS-CoV-2 infection among different individuals, and more cases need to be summarized.

## 4. Conclusion

In this case, the patient had a GATA2 mutation, which initially presented primarily as AML. After contracting SARS-CoV-2, the patient developed persistent immunodeficiency, leading to recurrent infections. These findings suggest that SARS-CoV-2 infection contributed to the occurrence of immunodeficiency in the patient. The treatment experience of this patient indicates that for AML patients with a GATA2 gene mutation, HSCT should be performed as early as possible after a suitable donor is identified.

## Author contributions

**Data curation:** Jinhua Chu, Linhai Yang.

**Investigation:** Zhengyu Wu, Anbang Wei, Yi Hong.

**Visualization:** Lingling Huang, Huaju Cai.

**Writing – original draft:** Jiale Cheng, Chengzhu Liu, Songji Tu.

**Writing – review & editing:** Jiale Cheng, Zhiwei Xie, Ningling Wang, Kunlong Zhang.
